# MicroWeaR: A new R package for dental microwear analysis

**DOI:** 10.1002/ece3.4222

**Published:** 2018-06-11

**Authors:** Flavia Strani, Antonio Profico, Giorgio Manzi, Diana Pushkina, Pasquale Raia, Raffaele Sardella, Daniel DeMiguel

**Affiliations:** ^1^ Dipartimento di Scienze della Terra Sapienza Università di Roma Rome Italy; ^2^ Istituto Italiano di Paleontologia Umana Rome Italy; ^3^ Dipartimento di Biologia Ambientale Sapienza Università di Roma Rome Italy; ^4^ Department of Geosciences and Geography University of Helsinki Helsinki Finland; ^5^ Dipartimento di Scienze della Terra Università di Napoli, Federico II Napoli Italy; ^6^ Fundación ARAID/Universidad de Zaragoza Zaragoza Spain; ^7^ Departamento de Ciencias de la Tierra Universidad de Zaragoza Zaragoza Spain; ^8^ Institut Català de Paleontologia Miquel Crusafont (ICP) Barcelona Spain

**Keywords:** diet reconstruction, open‐source software, paleoecology, R package, tooth microwear

## Abstract

Mastication of dietary items with different mechanical properties leaves distinctive microscopic marks on the surface of tooth enamel. The inspection of such marks (dental microwear analysis) is informative about the dietary habitus in fossil as well as in modern species. Dental microwear analysis relies on the morphology, abundance, direction, and distribution of these microscopic marks. We present a new freely available software implementation, *MicroWeaR*, that, compared to traditional dental microwear tools, allows more rapid, observer error free, and inexpensive quantification and classification of all the microscopic marks (also including for the first time different subtypes of scars). Classification parameters and graphical rendering of the output are fully settable by the user. *MicroWeaR* includes functions to (a) sample the marks, (b) classify features into categories as pits or scratches and then into their respective subcategories (large pits, coarse scratches, etc.), (c) generate an output table with summary information, and (d) obtain a visual surface‐map where marks are highlighted. We provide a tutorial to reproduce the steps required to perform microwear analysis and to test tool functionalities. Then, we present two case studies to illustrate how *MicroWeaR* works. The first regards a Miocene great ape obtained from through environmental scanning electron microscope, and other a Pleistocene cervid acquired by a stereomicroscope.

## 1 INTRODUCTION

Dental microwear analysis studies microscopic wear patterns produced on the occlusal enamel surfaces of teeth during mastication. It is one of the most valuable methods to assess dietary preferences in vertebrate taxa. Since the 1970s (see, among others, Gingerich, 1972; Grine, 1977; Puech, 1979; Walker, Hoeck, & Perez, 1978), microwear analysis has been successfully applied by anthropologists and paleontologists to gain insights into the diet of several extinct groups, such as primates, including humans and hominins (DeSantis, 2016; Scott et al., 2005; Teaford & Walker, 1984), ungulates (DeMiguel, Fortelius, Azanza, & Morales, 2008; Kaiser & Brinkmann, 2006; Mihlbachler, Campbell, Ayoub, Chen, & Ghani, 2016; Semprebon & Rivals, 2007; Solounias & Hayek, 1993; Solounias & Semprebon, 2002), and carnivores (Schubert, Ungar, & DeSantis, 2010; Van Valkenburgh, Teaford, & Walker, 1990). Dental microwear analysis relies on the microscopic marks on the occlusal surfaces of tooth enamel (and/or dentin), left by the food chewed by an individual up to a few hours, days, or weeks before its death—a phenomenon referred to as the “Last Supper effect”—, depending on the rate of turnover in dental microwear of a particular consumer and food (Grine, 1986). The abundance, morphology, size, distribution, and orientation of marks are a consequence of the mechanic abrasion produced by mastication and are distinctive between different diets, depending on the fracture properties of the food items. In ungulates, a higher number of scratches over pits indicate tough‐food (e.g., grasses) consumption. In contrast, a high number of pits indicate consumption of brittle, soft material such as leaves, fruits, and seeds (Solounias & Semprebon, 2002). In primates, a high occurrence of pits and coarse scratches is typical of hard‐object feeders (which primarily feed on nuts and roots, and unripe fruits). Conversely, diet rich in leaves and soft fruits, which is typical of folivorous and frugivorous primates, is characterized by a low percentage of pits and narrower scratches (King, Aiello, & Andrews, 1999; Teaford, 1988).

The most common way to observe and study enamel marks is using high definition, two‐dimensional pictures of a selected tooth crown region under either low or high magnification. The former, well‐established approach, known as Low magnification microwear (LMM), employs high‐precision casts of enamel surfaces observed by a standard stereomicroscope at 35× or 100× (for small mammals) magnification. Because it is fast and relatively low‐cost, LMM is probably the most common dental microwear method today (Bastl, Semprebon, & Nagel, 2012; Rivals & Athanassiou, 2008; Rodrigues, Merceron, & Viriot, 2009; Semprebon, Taob, Hasjanova, & Solounias, 2016; Solounias & Semprebon, 2002). High magnification microwear (HMM) relies instead on pictures obtained through scanning electron microscope (SEM; DeMiguel et al., 2008; Galbany, Martínez, & Pérez‐Pérez, 2004; King et al., 1999; Solounias, McGraw, Hayek, & Werdelin, 2000; Solounias & Moelleken, 1994), typically at 500× magnification. With environmental SEM (ESEM) devices, teeth can be observed directly without any damage, avoiding the risk of losing fine details during cast preparation. The downside of HMM is that it is more expensive and slower than LMM. Under both methods, enamel marks are classified, counted, and measured on a standard square area, whose size depends on the specific magnification adopted.

The recently introduced Dental microwear texture analysis (DMTA) (Merceron et al., 2009; Scott, Teaford, & Ungar, 2012; Scott et al., 2005; Ungar, Krueger, Blumenschine, Njau, & Scott, 2012) provides an alternative to both LMM and HMM. DMTA works with 3D surfaces and scale‐sensitive fractal data. Unlike the traditional methods, DMTA does not require the identification of any individual feature, and the analysis is automated, thus being faster and less affected by observer error than more traditional methods (Scott et al., 2005). However, DMTA is an expensive method, as it requires the use of white‐light scanning confocal microscopes (rather than simple 2D micrographs), and uses specific commercial software (Surfract^®^, ©2007; http://www.surfract.com/) and additional plugins (e.g., ToothFrax and SFrax) that increase the economic burden of the approach. Moreover, whereas traditional approaches record individual wear features to better understand individual morphologies and their orientations, DMTA focuses only on the overall pattern.

Both traditional (LMM and HMM) methods and DMTA require a software application to count and score enamel marks. Such software, except for *Microware* (Ungar, 1995), has never been specifically designed for microwear analysis and usually requires a costly license. In the case of *Microware*, one disadvantage is that it cannot discern between different subtypes of microscopic marks (e.g., large pits, coarse scratches). We therefore feel it is time to develop a freely available tool, specifically designed for microwear analysis, which allows for a more in‐depth and complete investigation of the tooth occlusal features.

Here, we introduce *MicroWeaR*, a new free, open‐access tool stored as an R package (Profico, Strani, Raia, & DeMiguel, 2018) that examines and scores microwear marks in a semiautomatic way. The method is designed to optimize sampling and classification of microscopic marks on high‐resolution pictures of tooth surfaces, under different magnification levels. Using a picture of a dental surface (provided with a metric reference for the definition of the scale factor) as the input, the operator defines the size and position of a working area first, and then tracks the microwear features. Each mark is automatically classified into one of the two main categories, either “scratch” or “pit.” It is important that, for each of these two categories, the tool recognizes two subcategories “small” and “large” pits, and “fine” and “coarse” scratches, and provides the user with summary statistics for each category and subcategory (count, mean, and standard deviation). We also provide *MicroWeaR* R code (R Development Core Team, 2009) along with the description of the application procedure. To illustrate the effectiveness of *MicroWeaR*, we further examined two case studies belonging to different taxonomic groups and different methodological procedures to obtain microwear information: a molar of the Miocene great ape *Anoiapithecus brevirostris* (see DeMiguel, Alba, & Moyà‐Solà, 2014) and a molar of the Pleistocene cervid *Cervus elaphus eostephanoceros* (Strani et al., 2018).

## 2 DESCRIPTION: *MICROWEAR* AS A TOOL FOR ESTIMATING MAMMAL DIETS

*MicroWeaR* has been developed to sample and semiautomatically classify multiple features from a picture at once. The tool functions (Table 1) support a variety of image file formats (i.e., “bmp,” “png,” “jpg,” and “tif”) and convert the input image into an .Ico object. The R code provides the user with an interactive plot to scale the .Ico object to its original size using a metric reference that should be embedded in the picture. For each microscopic feature sampling is achieved by recording two distances using the left‐click: the first one records the mark length, and the second its width. During the sampling procedure, the user may use the undo command to revert to a previous step and to zoom the picture in or out.

**Table 1:** List and descriptions of the functions embedded in the *MicroWeaR* R package

Function	Description
*class.Ico*	Convert an image into an object of class Ico. At present, the formats “jpeg,” “png,” and “tiff” are supported. Limited to grayscale images
*plot_Ico*	Plot an image of class Ico. Setting the matrix that contains the coordinates of the microwear marks as set, the function returns to the image
*scale_Ico*	Scale an image of Ico class by an interactive plot selecting two points on the metric reference and defining the length of the latter
*Warea.Ico*	Select a working area of an image of class Ico through an interactive plot. The operator has to select the center of the working area and its dimensions
*samp.traces*	Record detectable microwear marks through the interactive plot. *samp.traces* has an option to zoom in or out of the image of class Ico
*autom_class*	Classify the microwear marks in different subcategories as recorded by *samp.traces* (object type). The output also provides a matrix (object Matrix), where the length and the width in micron are reported for each mark. In addition, the image with recorded marks is produced
*cross.parallel*	Detect pairs of scratches, which are “parallel” or “crisscross”
*output.Ico*	Print a summary statistics table reporting the number of pits and scratches (and the size of any subcategory)
*mw.check*	Check (via interactive multi‐plot) the classification provided by the autom_class function. Before running *output.Ico* using the a posteriori classification, the user must run again *cross.parallel* using the updated microwear classification

At the end of the sampling session, the function *autom_class* provides an automatic classification of the marks as either pits or scratches. In turn, each pit is categorized as either “large” or “small” and each scratch is classified as either “fine” or “coarse.” Automatic classification parameters can also be set manually to customize the sampling procedure. The tool provides an additional function of direction to detect pairs of “parallel” and “crisscross” scratches. The *autom_class* function outputs a summary statistics table that can be exported in different format files (.txt, .sav for SPSS Statistics software, .csv for Excel spreadsheet), which includes the number of features of each type, the standard deviation and mean diameter of the pit, fine and coarse scratch lengths, and coarse scratch widths. Using the function *autom_class*, the user is able to save the original picture overlaid by a transparent layer of the identified microscopic marks highlighted with a distinctive, user‐defined color. The graphical rendering of the final output is itself fully customizable.

## 3 APPLICATION OF THE *MICROWEAR* PROCEDURE USING REAL CASE STUDIES

**Figure 1:**
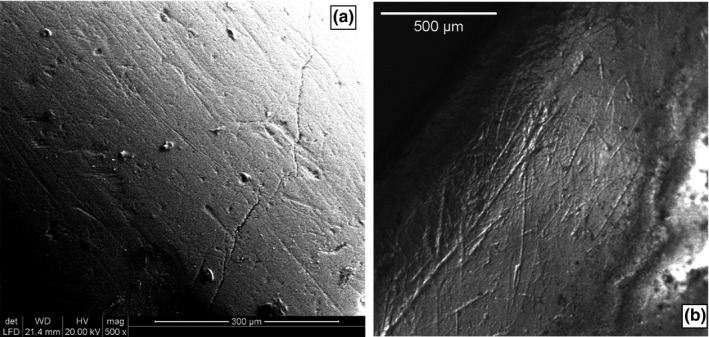
Enamel surface of the molars of *Anoiapithecus brevirostris* (a) and *Cervus elaphus eostephanoceros* (b)

We provide two case studies as examples of the step‐by‐step application of *MicroWeaR*. These are the enamel occlusal surfaces of a lower left second molar (m2) (“Phase II” crushing/grinding facet 9) of the Miocene great ape *A. brevirostris* (see DeMiguel et al., 2014) and an upper right first molar (M1) (antero‐lingual enamel band of the paracone) of the Middle Pleistocene cervid *C. e. eostephanoceros* (see Strani et al., 2018). The photomicrograph of the former was acquired through ESEM (at ×500 magnification) on the original specimen (Figure 1a), whereas the image of the latter was obtained using a stereomicroscope (×35 magnification) from a cast (Figure 1b). The mold and the cast of the molar tooth crown of *C. e. eostephanoceros* were prepared following standard procedures (Semprebon, Godfrey, Solounias, Sutherland, & Jungers, 2004; Solounias & Semprebon, 2002). The impression was made using high‐resolution Elite HD+ polysiloxane for the mold, and Araldite epoxy polymer for the cast. According to that, we provide microwear examples obtained from both high (×500) and low (×35) magnification and using either tooth originals or replicas. More comprehensive information on the taxa and the full description of the cleaning, molding/casting and examination procedures are available in DeMiguel et al. (2014) and Strani et al. (2018).

The *MicroWeaR* package supports the file formats “bmp,” “jpg,” “tif,” and “png.” As the first step, the *MicroWeaR* library is loaded into the R workspace. All the dependencies will be automatically installed or loaded as well. To begin the session, the user specifies the arguments *path* and *image.type* to import the image specifying where the file is located and its file format respectively.



require(devtools)
install_github(“MicroWeaR/MicroWeaR”,local=FALSE)
library(MicroWeaR)
library(zoom)
#load picture of *C. e. eostephanoceros*
data(C_el_pic)
#or load your picture typing:
#class.Ico(path, image.type = c(“jpg”, “png”, “tiff”))




The function *scale_Ico* scales the picture to the real size in micron (μm). The scaling procedure requires the selection of two points on the image. In a successive way, the operator will specify the scale length on the console.



#load scaled picture of *C. e. eostephanoceros*
data(C_el_sca)
#or scale your picture typing:
#scale_Ico(image.ico)




**Figure 2:**
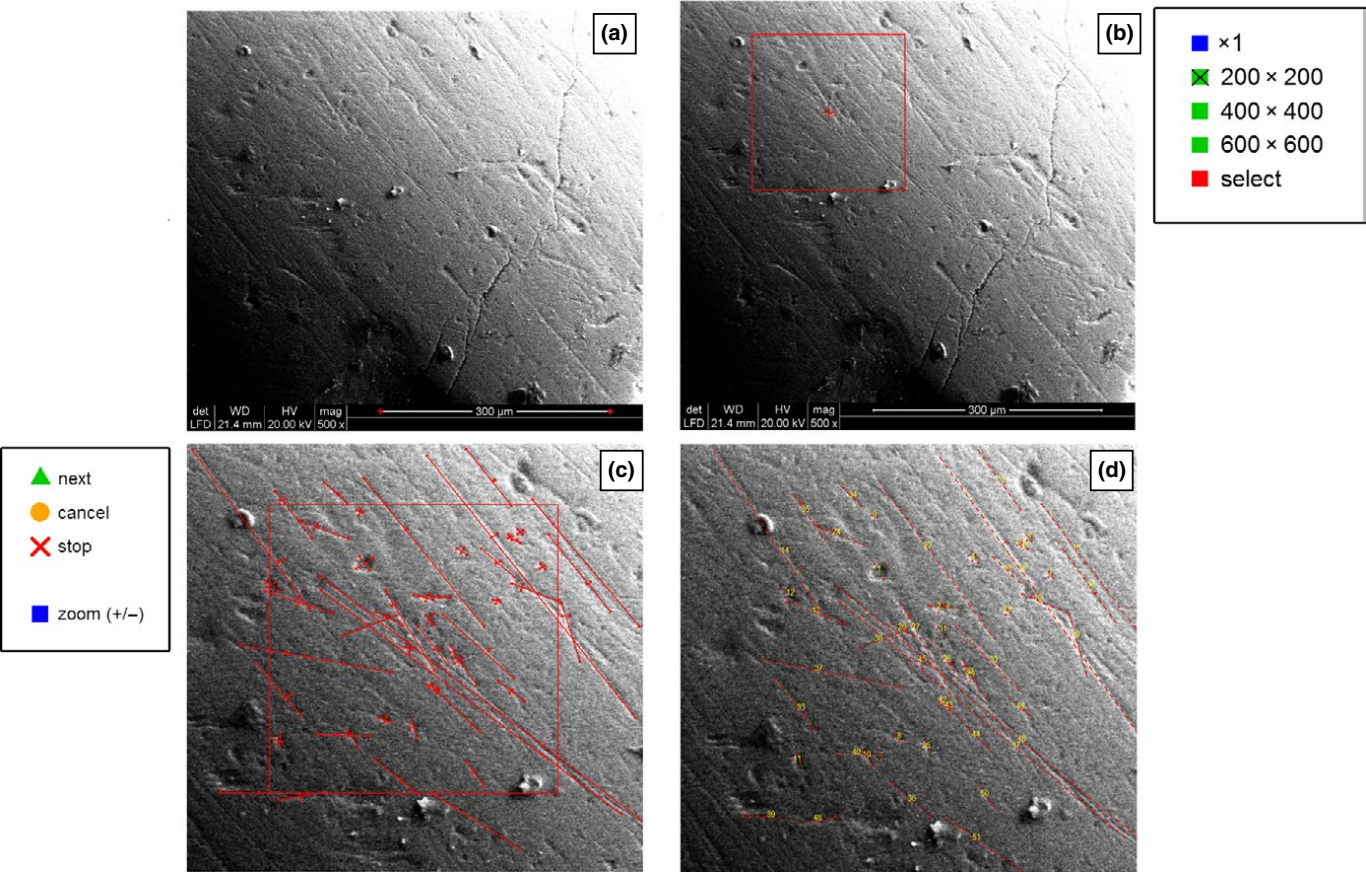
Step‐by‐step summary of semiautomatic enamel mark recognition performed using *MicroWeaR*. (a) Selection of two points on the reference metric scale to scale the image (top left). (b) Selection of the working area and size (“×1”: the size of the working area corresponds to the size of the input image; “select”: by selecting this option, the user can customize the size of the working area). (c) Sampling session (the “next” command allows to sample a new feature, the “cancel” command undoes the last sampling step, the “stop” command stops the sampling session, the “zoom” command allows to zoom in and out). (d) Sampled features displayed on the output image

After loading and scaling the image, the operator defines a working area (e.g., 200 × 200 μm) and a magnification factor to be applied. The argument *sizes* of the function *area.param* allows setting the default square working area size to be displayed in the interactive 2D plot during the sampling session. By default, either 200 × 200 μm, 400 × 400 μm or 600 × 600 μm working areas are selected, yet the user can define a custom area by choosing the “select” option and typing the desired size (side length) on the console (Figure 2a).
#load the selected working area
data(C_el_war)
#or select the working area typing:
#Warea.Ico(image.ico)



Once the working area is defined, the sampling session begins (Figure 2b). The operator defines four points for each mark: the first two record the mark length, and the last two its width (Figure 2c). 
#load the sampling session
data(C_el_sam)
#or start the sampling session typing:
#samp.traces(image.ico)



The arguments *cexp* and *lwdp* define the size and width of the points and lines of the marks, respectively. Considering that the image is scaled in micron, we suggest setting these parameters in respect to the dimension of the scaled picture, or inserting any other reasonable number (e.g., *cexp *= 50; *lwdp *= 1). In any case, if the *cexp* and the *lwdp* parameters are set as *NULL* the *samp.traces* function will adjust the values of these parameters automatically.

After the manual sampling, the tool automatically classifies each mark within one of the two categories of features: “scratch” and “pit” (Figure 2d). The classification is based on the length/width ratio; by default, this is set to 4 μm (≤4 for Pit and >4 for Scratch as proposed by Ungar, 1995). For each of these two categories, the tool recognizes different subcategories based on the diameter (for pits) and width (for scratches): “small” and “large” for pits (by default diameter ≤8 and >8 μm, respectively), and “fine” and “coarse” for scratches (by default the width ≤3 and <3 μm, respectively). All default discriminating values can be changed by the user in the *autom_class* function by editing the *Pit_Scr*,* Sm.Lg_pit* and *Fi.Co_Scr* arguments. 
# run type classification
class<‐autom_class(C_el_sam,C_el_war$image)
#or run the automatic classification typing:
#autom_class(big_matrix, image.ico, Pit_Scr = 4, Sm.Lg_Pit = 8, Fi.Co_Scr = 3)



The function *cross.parallel* calculates all the combinations of scratches and finds crossed and parallel scratch pairs. In detail, this function calculates the linear equation of the line passing through the two points that define the length of each mark. *MicroWeaR* uses the regression model parameters (intercepts and slopes) to classify scratch pairs as parallel (if the distance between the two scratches and their intersection point is greater than two‐times the square of the working area), or crisscross (if otherwise). In the latter case, the angle between intersecting scratches is calculated and produced in the output. 
scratches.ana<‐cross.parallel(big_matrix= C_el_sam,image.ico= C_el_war$image,Type=class$Type)



In addition, *MicroWeaR* provides a summary statistics report for each category and subcategory (including count, mean, and standard deviation) and the input picture with the sampled marks that can be exported in different file formats. Automatic classification parameters can also be manually edited and set allowing customizing each sampling session.

**Figure 3:**
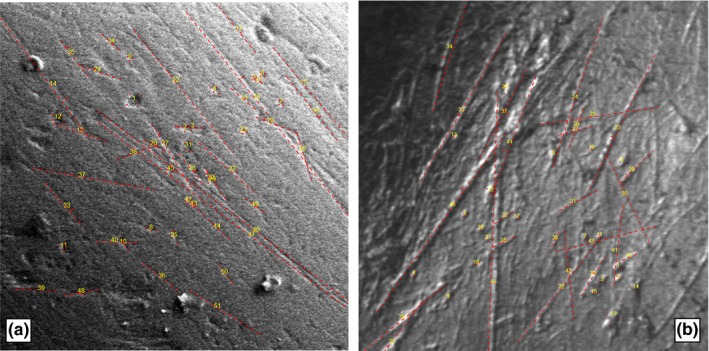
Final output images of *Anoiapithecus brevirostris* (a) and *Cervus elaphus eostephanoceros* (b). Microwear features were sampled on a 200 and a 400 μm^2^ area, respectively

At last, using the function *output.Ico* and specifying the matrix with the coordinates of the microwear marks, an image with the displayed marks is loaded as a plot (Figure 3). 
output.Ico(C_el_sam,class$Type,scratches.ana,C_el_war)



We provide a video tutorial as Supporting Information (Video S1) for the application of the tool in R environment.

### 3.1 Case studies interpretation

Regarding the occurrence of pits (*N* = 17), *A. brevirostris* resembles extant frugivores/mixed feeders such as *Cebus nigrivittatus*. It further displays somewhat wide scratches (Mean_width = 2.77 μm), in the range of *Pan troglodytes* (Mean_width = 2.6 μm) and *Pongo pygmaeus* (Mean_width = 2.8 μm), which suggests a certain degree of sclerocarpy. The results obtained by DeMiguel et al. (2014) show that, on average, *A. brevirostris* diet is somewhat intermediate in between *P*. *pygmaeus* and extant frugivores/mixed feeders such as *P. troglodytes* in terms of pitting incidence (*N* = 22), whereas it is similar to extant frugivores/mixed feeders in scratch width (Mean_width = 1.98 μm). These results confirm a soft‐fruit diet (albeit with some sclerocarpic components) and are fully consistent with those obtained using *MicroWeaR* (Table 2).

**Table 2:** Results of the microwear analysis applied to a tooth of *Anoiapithecus brevirostris*

	N.pits	N.sp	N.lp	%p	P	N.scratches	N.fs	N.cs	S	N.Ps	N.Xs	%Ps	%Xs
Count	17	9	8	33.3	425	34	20	14	850	62	9	85.3	26.5
Mean_length	7.64	5.29	9.73	/	/	20.94	22.38	18.87	/	/	/	/	/
Sd_length	3.75	1.06	4.08	/	/	19.24	23.14	12.21	/	/	/	/	/
Mean_width	2.86	2.54	3.14	/	/	2.77	1.13	5.12	/	/	/	/	/
Sd_width	1.95	1.54	2.31	/	/	2.41	1.41	1.35	/	/	/	/	/

*Note*

N.pits: number of pits; N.sp: number of small pits; N.lp: number of large pits; %p: percentage of pits; P: pits/mm^2^; N. scratches: number of scratches; N.fs: number of fine scratches; N.cs: number of coarse scratches; S: scratches/mm^2^; N.Ps: number of pairs of parallel scratches; N.Xs: number of scratches that cross each‐other; %Ps: percentage of parallel scratches; %Xs: percentage of scratches that cross each‐other.

The dental microwear pattern of the Pleistocene deer *C. e. eostephanoceros* has a similar amount of pits (*N* = 21) and scratches (*N* = 25) according to the *MicroWeaR* semiautomatic classification (Table 3). Most scratches are short and finely textured with a few long coarse scratches (Mean_length = 415.92 μm). Cross scratches are also detected (*N* = 15). Small pits are more abundant than larger ones (*N* = 13 and *N* = 8, respectively). A high number of pits and scratches with a prevalence of finely textured features indicates that *C. e. eostephanoceros* fed on a variety of plant types (both soft and abrasive), as commonly observed in modern mixed feeders (Solounias & Semprebon, 2002). The findings obtained using *MicroWeaR* are thus consistent with those obtained by Strani et al. (2018) where a larger, more indicative sample of *C. e. eostephanoceros* studied using both LMM and dental mesowear analysis, indicated a mixed feeder diet for this species.

**Table 3:** Results of the microwear analysis applied to a tooth of *Cervus elaphus eostephanoceros*

	N.pits	N.sp	N.lp	%p	P	N.scratches	N.fs	N.cs	S	N.Ps	N.Xs	%Ps	%Xs
Count	21	13	8	45.7	131	25	17	8	156	4	15	20.0	36.0
Mean_length	20.38	11.96	34.06	/	/	240.52	157.98	415.92	/	/	/	/	/
Sd_length	14.52	5.79	14.11	/	/	178.58	108	176	/	/	/	/	/
Mean_width	4.52	2.73	7.43	/	/	1.66	0.73	3.62	/	/	/	/	/
Sd_width	4.73	2.24	6.3	/	/	2.36	0.93	3.25	/	/	/	/	/

*Note*

N.pits: number of pits; N.sp: number of small pits; N.lp: number of large pits; %p: percentage of pits; P: pits/mm^2^; N. scratches: number of scratches; N.fs: number of fine scratches; N.cs: number of coarse scratches; S: scratches/mm^2^; N.Ps: number of pairs of parallel scratches; N.Xs: number of scratches that crosses each‐other. %Ps: percentage of parallel scratches; %Xs: percentage of scratches that cross each‐other.

## 4 SIGNIFICANCE OF THE TOOL

Using traditional LMM and HMM methods, one key factor affects the validity of the results, that is how different operators count and discriminate among microscopic marks (DeSantis et al., 2013; Mihlbachler, Beatty, Caldera‐Siu, Chan, & Lee, 2012). The use of a semiautomatic approach minimizes the intraobserver error because the only manual step in the whole procedure is the definition of the initial and the end point of each enamel mark. The automatic differentiation between subcategories also helps to reduce interobserver error rates when it comes to detailed interpretation of microwear features, which are usually high with traditional semiautomatic approaches (Galbany et al., 2005; Grine, Ungar, & Teaford, 2002; Mihlbachler et al., 2012). Given that *MicroWeaR* can be used for the analysis of any 2D image containing scars, it is also useful for recording lineal striations (i.e., number, length and breadth of scratches) in micrographs taken on nonocclusal tooth surfaces and, therefore, extensible to buccal enamel microwear quantification (Galbany & Pérez‐Pérez, 2004; Pérez‐Pérez, Lalueza, & Turbón, 1994; Puech, 1981) as well.

Since the creation of the R platform, libraries addressing natural science applications have rapidly increased (R Core Team, 2000). The open‐access nature of the R platform allows tools to be rapidly improved, by introducing new functionalities that are under immediate diffusion and testing through the R community. According to that, we designed *MicroWeaR* in order to work under different operating systems (i.e., Windows, OSX, Linux).

**Figure 4:**
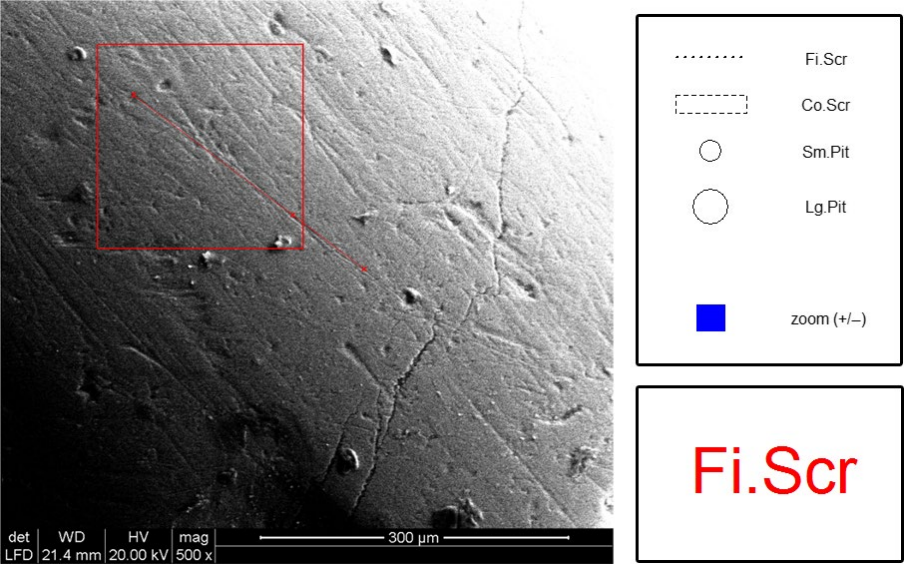
Additional *MicroWeaR* functionality: classification editing. The automatic classification of each mark can be manually edited at the end of the procedure using a multiplots interactive interface. Co.Scr: coarse scratch; Fi.Scr: fine scratch; Lg.Pit: large pit; Sm.Pit: small pit

*MicroWeaR* allows the automatic classification of the marks left on the enamel surface by the last foods (Grine, 1986) processed. Such automaticity helps keeping inter‐ and intraobserver error low (categories automatically assigned to each mark can be nonetheless manually edited using the *mw.check* function; Figure 4) and makes the dental microwear analysis faster, more robust, and cheaper than with any other comparable application.

## 5 CONCLUSIONS

A new software implementation for dental microwear analysis, *MicroWeaR*, offers a semiautomatic open‐access tool for quantification and classification of the microscopic enamel marks, stored as an R package. *MicroWeaR* is less time‐consuming and less prone to observer errors in comparison with the conventional microwear analysis with two‐dimensional imaging methods (LMM, HMM), as it is inexpensive compared to a new three‐dimensional method (DMTA). It works for any 2D image containing microwear scars. Thus, it is useful for the quantification of marks as observed under either high or low magnification, on both occlusal and nonocclusal (e.g., buccal) tooth surfaces (dentin or enamel), and from either tooth originals or replicas. *MicroWeaR* is designed to work in different operating systems (e.g., Windows, OSX, Linux) and due to its intrinsic characteristics, it is unique to be developed further.

## CONFLICT OF INTEREST

None declared.

## AUTHORS' CONTRIBUTIONS

F.S., A.P., P.R., and D.DM. conceived the ideas and designed methodology; D.DM. and F.S. collected the data; F.S. and A.P. wrote the R code with the contribution of P.R. and D.DM.; F.S., A.P., P.R., and D.DM. led the writing of the manuscript and contributed to the implementation of example analyses. D.P., R.S., and G.M. contributed helpful comments and provided inputs for the manuscript. All authors revised the manuscript and gave final approval.

## AVAILABILITY AND DATA ACCESSIBILITY

*MicroWeaR can be downloaded from* https://github.com/MicroWeaR (https://doi.org/10.5281/zenodo.1233505). We encourage authors to cite Strani et al. (this paper) if you use *MicroWeaR* for research, education, and outreach. As an application designed to be part of R, *MicroWeaR* is available as a package to run on different operating systems (Windows, Mac OS or Linux).

The results reported in this paper were obtained using *MicroWeaR* R package. The code and real examples of use are available in the *MicroWeaR* R package.

## References

[ece34222-bib-0001] Bastl, K. , Semprebon, G. , & Nagel, D. (2012). Low‐magnification microwear in Carnivora and dietary diversity in Hyaenodon (Mammalia: Hyaenodontidae) with additional information on its enamel microstructure. Palaeogeography, Palaeoclimatology, Palaeoecology, 348–349, 13–20. 10.1016/j.palaeo.2012.05.026

[ece34222-bib-0002] DeMiguel, D. , Alba, D. M. , & Moyà‐Solà, S. (2014). Dietary specialization during the evolution of Western Eurasian hominoids and the extinction of European Great Apes. PLoS One, 9, e97442 10.1371/journal.pone.0097442 24848272 PMC4029579

[ece34222-bib-0003] DeMiguel, D. , Fortelius, M. , Azanza, B. , & Morales, J. (2008). Ancestral feeding state of ruminants reconsidered: Earliest grazing adaptation claims a mixed condition for Cervidae. BMC Evolutionary Biology, 8, 1–13. 10.1186/1471-2148-8-13 18205907 PMC2270802

[ece34222-bib-0004] DeSantis, L. R. G. (2016). Dental microwear textures: Reconstructing diets of fossil mammals. Surface Topography: Metrology and Properties, 4, 023002 10.1088/2051-672x/4/2/023002

[ece34222-bib-0005] DeSantis, L. R. G. , Scott, J. R. , Schubert, B. W. , Donohue, S. L. , McCray, B. M. , Van Stolk, C. A. , … O'Hara, M. C. (2013). Direct comparisons of 2D and 3D dental microwear proxies in extant herbivorous and carnivorous mammals. PLoS One, 8, e71428 10.1371/journal.pone.0071428 23936506 PMC3735535

[ece34222-bib-0006] Galbany, J. , Martínez, L. , López‐Amor, H. , Espurz, V. , Hiraldo, O. , Romero, A. , … Pérez‐Pérez, A. (2005). Error rates in buccal‐dental microwear quantification using scanning electron microscopy. Scanning, 27(1), 23–29. 10.1002/sca.4950270105 15712754

[ece34222-bib-0007] Galbany, J. , Martínez, L. M. , & Pérez‐Pérez, A. (2004). Tooth replication techniques, SEM imaging and microwear analysis in primates: Methodological obstacles. Anthropologie, 42(1), 5–6.

[ece34222-bib-0008] Galbany, J. , & Pérez‐Pérez, A. (2004). Buccal enamel microwear variability in Cercopithecoidea primates as a reflection of dietary habits in forested and open savanna environments. Anthropologie, 42(1), 13–19.

[ece34222-bib-0009] Gingerich, P. D. (1972). Molar occlusion and jaw mechanics of the Eocene primate *Adapis* . American Journal of Physical Anthropology, 36, 359–368. 10.1002/ajpa.1330360306 5064309

[ece34222-bib-0010] Grine, F. E. (1977). Analysis of early hominid deciduous molar wear by scanning electron microscopy: A preliminary report. Proceedings of the Electron Microscopy, Society of South Africa, 7, 157–158.

[ece34222-bib-0011] Grine, F. E. (1986). Dental evidence for dietary differences in *Australopithecus* and *Paranthropus*: A quantitative analysis of permanent molar microwear. Journal of Human Evolution, 15, 783–822. 10.1016/S0047-2484(86)80010-0

[ece34222-bib-0012] Grine, F. E. , Ungar, P. S. , & Teaford, M. F. (2002). Error rates in dental microwear quantification using scanning electron microscopy. Scanning, 24(3), 144–153. 10.1002/sca.4950240307 12074496

[ece34222-bib-0013] Kaiser, T. M. , & Brinkmann, G. (2006). Measuring dental wear equilibriums—The use of industrial surface texture parameters to infer the diets of fossil mammals. Palaeogeography, Palaeoclimatology, Palaeoecology, 239(3), 221–240. 10.1016/j.palaeo.2006.01.013

[ece34222-bib-0014] King, T. , Aiello, L. C. , & Andrews, P. (1999). Dental microwear of *Griphopithecus alpani* . Journal of Human Evolution, 36(1), 3–31. 10.1006/jhev.1998.0258 9924132

[ece34222-bib-0015] Merceron, G. , Scott, J. , Scot, t. R. S. , Geraads, D. , Spassov, N. , & Ungar, P. S. (2009). Folivory or fruit/seed predation for *Mesopithecus*, an earliest colobine from the late Miocene of Eurasia? Journal of Human Evolution, 57, 732–738. 10.1016/j.jhevol.2009.06.009 19733899

[ece34222-bib-0016] Mihlbachler, M. C. , Beatty, B. L. , Caldera‐Siu, A. , Chan, D. , & Lee, R. (2012). Error rates and observer bias in dental microwear analysis using light microscopy. Palaeontologia Electronica, 15(1), 12A, 1–22.

[ece34222-bib-0017] Mihlbachler, M. C. , Campbell, D. , Ayoub, M. , Chen, C. , & Ghani, I. (2016). Comparative dental microwear of ruminant and perissodactyl molars: Implications for paleodietary analysis of rare and extinct ungulate clades. Paleobiology, 42(1), 98–116. 10.1017/pab.2015.33

[ece34222-bib-0018] Pérez‐Pérez, A. , Lalueza, C. , & Turbón, D. (1994). Intraindividual and intragroup variability of buccal tooth striation pattern. American Journal of Physical Anthropology, 94, 175–187. 10.1002/ajpa.1330940203 8085610

[ece34222-bib-0019] Profico, A. , Strani, F. , Raia, P. , & DeMiguel, D. (2018). MicroWeaR R package, (Version 0.99). Zenodo. Retrieved from https://github.com/MicroWeaR/MicroWeaR. 10.5281/zenodo 1233505.

[ece34222-bib-0020] Puech, P. F. (1979). The diet of early man: Evidence from abrasion of teeth and tools. Current Anthropology, 20, 590–592. 10.1086/202335

[ece34222-bib-0021] Puech, P. F. (1981). Tooth wear in La Ferrassie man. Current Anthropology, 22, 424–430. 10.1086/202699

[ece34222-bib-0022] R Core Team (2000). R language definition. Vienna, Austria: R Foundation for Statistical Computing.

[ece34222-bib-0500] R Development Core Team (2009). R: A Language and Environment for Statistical Computing. R Foundation for Statistical Computing, Vienna. http://www.R-project.org.

[ece34222-bib-0023] Rivals, F. , & Athanassiou, A. (2008). Dietary adaptations in an ungulate community from the late Pliocene of Greece. Palaeogeography, Palaeoclimatology, Palaeoecology, 265, 134–139. 10.1016/j.palaeo.2008.05.001

[ece34222-bib-0024] Rodrigues, H. G. , Merceron, G. , & Viriot, L. (2009). Dental microwear patterns of extant and extinct Muridae (Rodentia, Mammalia): Ecological implications. Naturwissenschaften, 96, 537–542. 10.1007/s00114-008-0501-x 19127354

[ece34222-bib-0025] Schubert, B. W. , Ungar, P. S. , & DeSantis, L. R. G. (2010). Carnassial microwear and dietary behaviour in large carnivorans. Journal of Zoology, 280, 257–263. 10.1111/j.1469-7998.2009.00656.x

[ece34222-bib-0026] Scott, R. S. , Teaford, M. F. , & Ungar, P. S. (2012). Dental microwear texture and anthropoid diets. American Journal of Physical Anthropology, 147, 551–579. 10.1002/ajpa.22007 22331579

[ece34222-bib-0027] Scott, R. S. , Ungar, P. S. , Bergstrom, T. S. , Brown, C. A. , Grine, F. E. , Teaford, M. F. , & Walker, A. (2005). Dental microwear texture analysis shows within‐species diet variability in fossil hominins. Nature, 436(7051), 693–695. 10.1038/nature03822 16079844

[ece34222-bib-0028] Semprebon, G. , Godfrey, L. , Solounias, N. , Sutherland, M. , & Jungers, W. (2004). Can low‐magnification stereomicroscopy reveal diet? Journal of Human Evolution, 47, 115–144. 10.1016/j.jhevol.2004.06.004 15337412

[ece34222-bib-0029] Semprebon, G. M. , & Rivals, F. (2007). Was grass more prevalent in the pronghorn past? An assessment of the dietary adaptations of Miocene to recent Antilocapridae (Mammalia: Artiodactyla). Palaeogeography, Palaeoclimatology, Palaeoecology, 253, 332–347. 10.1016/j.palaeo.2007.06.006

[ece34222-bib-0030] Semprebon, G. M. , Taob, D. , Hasjanova, J. , & Solounias, N. (2016). An examination of the dietary habits of *Platybelodon grangeri* from the Linxia Basin of China: Evidence from dental microwear of molar teeth and tusks. Palaeogeography, Palaeoclimatology, Palaeoecology, 457, 109–116. 10.1016/j.palaeo.2016.06.012

[ece34222-bib-0031] Solounias, N. , & Hayek, L. A. C. (1993). New methods of tooth microwear analysis and application to dietary determination of two extinct antelopes. Journal of Zoology, 229(3), 421–445. 10.1111/j.1469-7998.1993.tb02646.x

[ece34222-bib-0032] Solounias, N. , McGraw, W. S. , Hayek, L. A. , & Werdelin, L. (2000). The paleodiet of the Giraffidae In Vrba E. S. & Schaller G. B. (Eds.), Antelopes, deer, and relatives: Fossil record, behavioural ecology, systematics, and conservation (pp. 84–95). New Haven, CT: Yale University Press.

[ece34222-bib-0033] Solounias, N. , & Moelleken, S. M. C. (1994). Dietary differences between two archaic ruminant species from Sansan, France. Historical Biology, 7, 203–220. 10.1080/10292389409380454

[ece34222-bib-0034] Solounias, N. , & Semprebon, G. (2002). Advances in the reconstruction of ungulate ecomorphology with application to early fossil equids. American Museum Novitates, 3366, 1–49. 10.1206/0003-0082(2002)366&lt;0001:AITROU&gt;2.0.CO;2

[ece34222-bib-0035] Strani, F. , DeMiguel, D. , Bona, F. , Sardella, R. , Biddittu, I. , Bruni, L. , … Bellucci, L. (2018). Ungulate dietary adaptations and palaeoecology of the Middle Pleistocene site of Fontana Ranuccio (Anagni, Central Italy). Palaeogeography, Palaeoclimatology, Palaeoecology, 496, 238–247. 10.1016/j.palaeo.2018.01.041

[ece34222-bib-0036] Teaford, M. F. (1988). A review of dental microwear and diet in modern mammals. Scanning Microscopy, 2, 1149–1166. 3041572

[ece34222-bib-0037] Teaford, M. F. , & Walker, A. (1984). Quantitative differences in dental microwear between primate species with different diets and a comment on the presumed diet of *Sivapithecus* . American Journal of Physical Anthropology, 64, 191–200. 10.1002/ajpa.1330640213 6380302

[ece34222-bib-0038] Ungar, P. S. (1995). A semiautomated image analysis procedure for the quantification of dental microwear II. Scanning, 17, 57–59. 10.1002/sca.4950170108 7704317

[ece34222-bib-0039] Ungar, P. S. , Krueger, K. L. , Blumenschine, R. J. , Njau, J. K. , & Scott, R. S. (2012). Dental microwear texture analysis of hominins recovered by the Olduvai Landscape Paleoanthropology Project, 1995–2007. Journal of Human Evolution, 63, 429–437. 10.1016/j.jhevol.2011.04.006 21784504

[ece34222-bib-0040] Van Valkenburgh, B. , Teaford, M. F. , & Walker, A. (1990). Molar microwear and diet in large carnivores*:* Inferences concerning diet in the sabretooth cat, *Smilodon fatalis* . Journal of Zoology, 222, 319–340. 10.1111/j.1469-7998.1990.tb05680.x

[ece34222-bib-0041] Walker, A. , Hoeck, H. N. , & Perez, L. (1978). Microwear of mammalian teeth as an indicator of diet. Science, 201, 908–910. 10.1126/science.684415 684415

